# Nanophotonic resonator assisted photonic spin Hall enhancement for sensing application

**DOI:** 10.1038/s41598-023-36417-5

**Published:** 2023-06-07

**Authors:** Amit Kumar Goyal, Divyanshu Divyanshu, Yehia Massoud

**Affiliations:** Innovative Technologies Laboratories (ITL), King Abdullah University of Science and Technology (KAUST), 23955 Thuwal, Saudi Arabia

**Keywords:** Optical sensors, Photonic crystals, Nanophotonics and plasmonics

## Abstract

This manuscript presents a dielectric resonator structure with altered dispersion characteristics to enhance the photonic spin Hall effect (PSHE). The structural parameters are optimized to enhance the PSHE at 632.8 nm operating wavelength. The thickness-dependent angular dispersion analysis is carried out to optimize the structure and obtain the exceptional points. The PSHE-induced spin splitting shows a high sensitivity to the optical thickness of the defect layer. This gives a maximum PSHE-based transverse displacement (PSHE-TD) of around 56.66 times the operating wavelength at an incidence angle of 61.68°. Moreover, the structure’s capability as a PSHE-based refractive index sensor is also evaluated. The analytical results demonstrate an average sensitivity of around 33,720 μm/RIU. The structure exhibits around five times higher PSHE-TD and approximately 150% improvement in sensitivity than the recently reported values in lossy mode resonance structures. Due to the purely dielectric material-assisted PhC resonator configurations and significantly higher PSHE-TD, the development of low-cost PSHE-based devices for commercial applications is envisaged.

## Introduction

Spin-orbit interaction (SOI) is a fundamental occurrence observed in various scientific research areas like condensed matter physics, spintronics, and photonics. In recent years, much interest has grown in investigating the spin Hall effect (SHE) in electrons, which is a collection of relativistic SOI phenomenon^1^. The ability to generate, manipulate, and detect spin-currents has given rise to applications such as boolean logic, memories, computing, and hardware security^2–4^ etc. Similarly, the photonic spin Hall effect (PSHE) has shown various promising applications and is expected to show superior performance owing to its inherent advantage. The PSHE refers to the spin-dependent transverse shift of photons with respect to the geometric optical trajectory when the beam passes through an optical interface or inhomogeneous medium^5,6^. Bliokh et al. in the year 2004 introduced the topological spin-based splitting of photons in the inhomogeneous medium using the concept of geometric-Berry phase (GBP)^7,8^. Onoda et al. in the same year, proposed the presence of PSHE based on GBP and optical angular momentum (OAM) conversation^9^ and further proposed a comprehensive theoretical approach for calculating the PSHE in the year 2007^10^. Thus, the origin of PSHE is associated with the SOI of light, OAM, and geometric phases, i.e., Rytov-Vlasimirskii phase and Pancharatnam-Berry phase^11^. Due to the PSHE effect, the reflected beam splits into corresponding polarization states (RCP/LCP or H/V polarization).

The first experimental demonstration of the PSHE was conducted in 2008 by Hosten et al. at an air-glass interface^12^. Following this, PSHE investigation has been carried out in chiral materials^13^, metallic thin films^14^, topological materials^15^, two-dimensional atomic crystals^16^, metamaterials^17^ and, Photonic crystals (PhC)^18^, etc. Here, the major emphasis is to enhance the PSHE, which has been investigated considering various nanophotonic techniques such as Brewster angle^19^, Surface Plasmon Resonance (SPR)^20–22^, optical pumping^23^ and, lossy mode resonance (LMR)^24^, etc . These techniques have been utilized in designing highly sensitive refractive index sensors using PSHE^14,24–27^. However, the reported PSHE-TD is very low in most reported structures, limiting its widespread use in various exciting applications. The PSHE-TD can also be enhanced considering multilayered photonic crystal-based nano-devices because of their light-controlling properties^28^. These devices have witnessed considerable growth in demand in various exciting applications over the last few years, including biomedical diagnostics, liquid/gas sensing, and environmental monitoring^29,30^. These nanostructures can be optimized to manipulate light-matter interactions, suppressing a particular polarization. This property enhances PSHE and thus shows its capabilities in several interesting applications in a wide range of scientific areas^31^. However, to our knowledge, work has yet to be reported in the literature about only dielectric material-assisted PhC resonator configurations for refractive index sensing using PSHE enhancement.

This research proposes a novel dielectric PhC resonator structure optimization toward PSHE-TD enhancement. The proposed design comprises a bilayer-PhC structure of silicon nitride and silicon dioxide materials. The structural parameters are optimized and regulated to alter the dispersion characteristics, which results in a very low or negligible reflection for a particular polarization (here, *p*-polarization) and a high reflection for another (here, *s*-polarization). This gives a very high ratio of Fresnel reflection coefficient for *s*, and *p*-polarized light, i.e., $\left| r_s\right| /\left| r_p\right|$, which is essential in obtaining a high value of PSHE-TD^18^. The structure response and corresponding PSHE-TD are analyzed over a wider incidence angle from 0° to 90°. The impact of varying defect layer thickness on the generation of PSHE-TD is analyzed thoroughly. The analytical results demonstrate an enhanced PSHE-TD of 56.66 times the operational wavelength at 124.16 nm defect layer thickness for a 61.68° of incidence angle. Finally, the structure’s PSHE-based refractive index sensor capability is also demonstrated. The angular interrogation gives an average sensitivity of around 33,720 μm/RIU. The proposed structure shows a $\approx$ 150% higher sensitivity than recently reported LMR based structure^24^. Finally, the structural performance is also compared with recently reported values. The proposed device is advantageous as the same structure can be optimized to generate PSHE for both horizontal and vertical polarization at user-defined wavelengths. Therefore, the proposed device provides a high-performance PSHE-based sensor for medical and commercial applications having a very simple structure, easy fabrication, and low cost.

The paper is organized into three major sections. The theoretical model and device structure of the nanophotonic resonator used in this work is discussed in the “Device structure and modeling” section. The effect of defect layer thickness variation on PSHE shift is discussed in the “Results and discussion” section, and finally, the last section provides the “Conclusion”.

## Device structure and modeling

**Figure 1:**
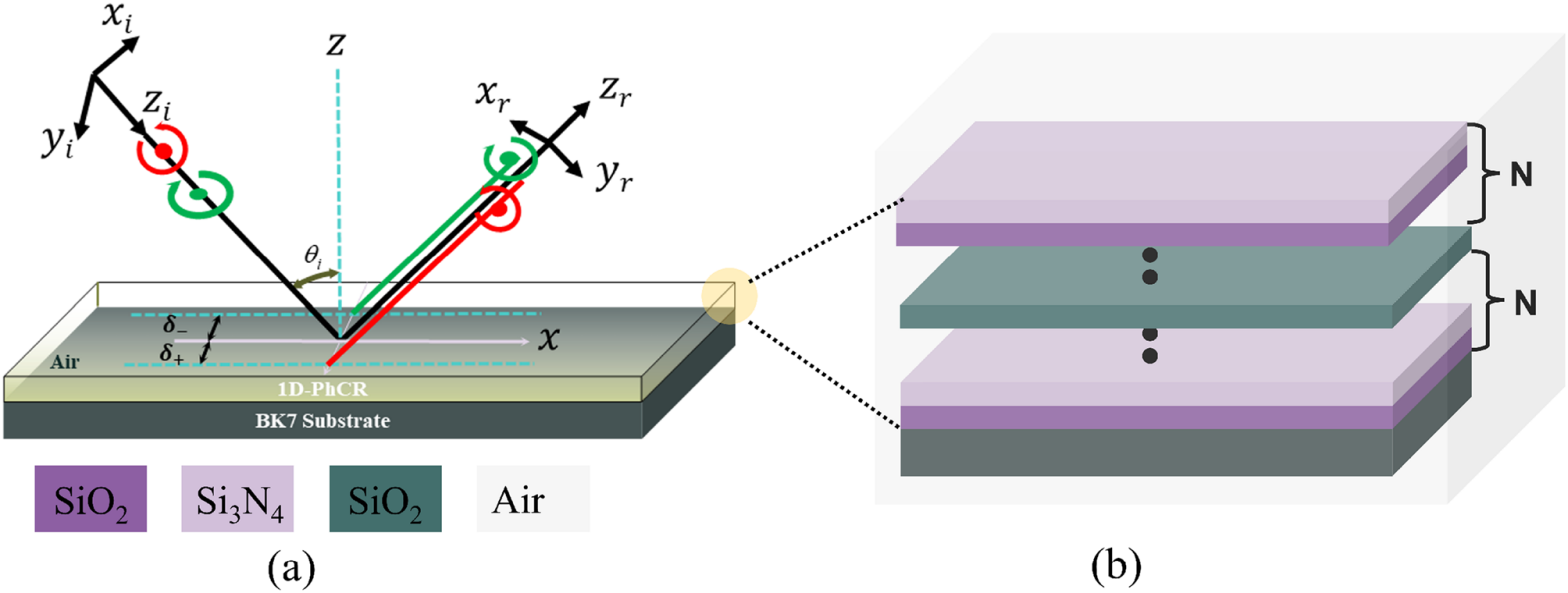
(**a**) Schematic representation of PSHE in nanophotonic structures and, (**b**) proposed device structure with configuration: [substrate$\mid$(A,B)$^N \mid$D$\mid$(A,B)$^N \mid$Air], with N=10.

The schematical representation of the PSHE effect and corresponding splitting of photons is shown in Fig. 1a. Here, $\hbox {Z}_{i}$ and $\hbox {Z}_{r}$ are the incident and reflected fields at the nanophotonic structure’s top interface, $\delta _{\pm }$ represents the PSHE’s transverse displacement (PSHE-TD), and $\theta _{i}$ is the incidence angle. Whereas, Fig. 1b provides the proposed 1D-PhC resonator structure having [Substrate$\mid$(A,B)$^N \mid$D$\mid$(A,B)$^N \mid$Air] configuration. The structure is designed considering ‘BK7 Glass’ as a substrate (refractive index 1.515 and extinction coefficient of 4.09 $\times$ $10^{-7}$). Here, ‘AB’ represents a unit cell having $\text {SiO}_{\text {2}}$ as a material ‘A’ and, $\text {Si}_{\text {3}} \text {N}_{\text {4}}$ as material ‘B’. The unit cell is repeated ‘N’ (here, 10) times to obtain a sufficient higher reflectance (here, > 99%). The defect layer ‘D’ is considered as $\text {SiO}_{\text {2}}$ (similar to ‘A’ for simplicity). The materials A($n_{L}$) and B($n_{H}$) possess refractive indices of 1.46 and 2.2, which are calculated using the Sellmeier equation. The ‘A and B’ material’s inherent loss is accounted for by taking the imaginary dielectric constant as 0.0001 and 0.0007, respectively. The physical thickness of the materials is calculated considering the Quarter wavelength Bragg stack configuration. Thus the thickness of A($D_{l}$) and B($D_{h}$) were selected as 128 nm and 85 nm, respectively. Initially, the defect layer thickness ($D_{d}$) is considered equivalent to layer ‘A’.

Considering an incident monochromatic Gaussian beam whose angular spectrum is given by Eq. (1) with an operating wavelength of $\lambda$ and beam waist $w_{0}$,

1 $$\begin{aligned} \tilde{\textbf{E}}_{i \pm }=\left( \textbf{e}_{i x} \pm i\textbf{e}_{i y}\right) \frac{w_0}{\sqrt{2 \pi }} \exp \left[ -\frac{w_0^2\left( k_{i x}^2+k_{i y}^2\right) }{4}\right] , \end{aligned}$$

where, $k_{ix}$ and $k_{iy}$ are components of wave-vector in the $x_{i}$ and $y_{i}$ direction and +/- designate left/right circular polarization components, respectively.

A linear polarized Gaussian beam is divided into two circularly polarized components with opposite shifts due to the spin Hall effect, i.e., the PSHE is described for the spin-dependent splitting of left- and right-handed circularly polarized components, and thus the reflected field centroid should be calculated by considering the shifts of light beam centroid compared to the geometrical-optics prediction. As the Gaussian beam can be formulated as a localized wave packet whose spectrum is arbitrarily narrow, they have been widely used for PSHE-based studies^19–21^.

The spin-basis set representation of Eq. (1) is described in Eq. (2),

2 $$\begin{aligned} \tilde{\textbf{E}}_i^H=\left( \tilde{\textbf{E}}_{i+}+\tilde{\textbf{E}}_{i-}\right) / \sqrt{2}, \tilde{\textbf{E}}_i^V=i\left( \tilde{\textbf{E}}_{i-}-\tilde{\textbf{E}}_{i+}\right) / \sqrt{2} \end{aligned}$$

here *H*(*V*) represent the horizontal (vertical) polarization state. The relation between the reflected angular spectrum and incident angular spectrum needs to be ascertained to obtain the required PSHE-TD. The desired relation is obtained by co-ordinate rotation described in Eq. (3)^19^,

3 $$\begin{aligned} {\tilde{\textrm{E}}}_r\left( k_{r x}, k_{r y}\right) ={\textrm{R}}_M {\tilde{\textrm{E}}}_i\left( k_{i x}, k_{i y}\right) \end{aligned}$$

here,

$$\begin{aligned} \textrm{R}_M=\left[ \begin{array}{cc} r_p &{} \frac{k_{r y} \cot \theta _i\left( r_s+r_p\right) }{k} \\ -\frac{k_{r y} \cot \theta _i\left( r_s+r_p\right) }{k} &{} r_s \end{array}\right] \end{aligned}$$

where, *k* = 2$\pi$/$\lambda$ is the wave number, Fresnel reflection coefficient is represented by $r_{s,p}$ for the corresponding polarization states.

In terms of *H* polarization states, based on Eqs. (2, 3), the reflected angular spectrum is described by Eq. (4),

4 $$\begin{aligned} \tilde{\textrm{E}}_r^H=\frac{r_p}{\sqrt{2}}\left[ \exp \left( +i k_{r y} \Delta _r^H\right) \tilde{\textrm{E}}_{r+}+\exp \left( -i k_{r y} \Delta _r^H\right) \tilde{\textrm{E}}_{r-}\right] \end{aligned}$$

here,

$$\Delta _r^H=\left( 1+\frac{r_s}{r_p}\right) \cot \theta _i / k$$

Similar steps would yield reflected angular spectrum for *V* polarized reflected spectrum, i.e., $\tilde{\textrm{E}}_r^V$. To obtain the generalized Fresnel reflection coefficient expression, conventional numerical methods like the transfer matrix method (TMM) can be used^32^. For such multi-layer structure 2*2 transmission matrix method can give the desired expression^33^:

$$\begin{aligned} Z=T_{1 \rightarrow 2} P_2 T_{2 \rightarrow 3} P_3 T_{3 \rightarrow 4} \cdots P_{l-1} T_{l-1 \rightarrow l} \end{aligned}$$

here $$T_{l-1 \rightarrow l}=\frac{1}{t_{l-1 \rightarrow l}}\left[ \begin{array}{cc}1 &{} r_{l-1 \rightarrow l} \\ r_{l-1 \rightarrow l} &{} 1\end{array}\right] $$ represents the transmission matrix from $(l-1)th$ layer to *lth* layer.

$P_l=$ $$\left[ \begin{array}{cc}\exp \left( i k_{l z} d_t\right) &{} 0 \\ 0 &{} \exp \left( -i k_{l z} d_t\right) \end{array}\right] $$ is the propagation matrix for *lth* layer with thickness $d_t$. The reflection coefficient is given by TMM elements as^33^:

$$\begin{aligned} r_{s, p}=\frac{Z_{21}}{Z_{11}} \end{aligned}$$

To obtain the $r_{s, p}$ Taylor-series expansion is used which expands the Fresnel coefficients as:

5 $$\begin{aligned} r_{s, p}\left( k_{i x}\right) =r_{s, p}\left( k_{i x}=0\right) +k_{i x}\left[ \frac{\partial _{r_{s, p}\left( k_{i x}\right) }}{\partial k_{i x}}\right] _{k_{i x}=0}+\sum _{j=2}^N \frac{k_{i x}{ }^N}{j !}\left[ \frac{\partial j_{r_{s, p}}\left( k_{i x}\right) }{\partial k_{i x}{ }^j}\right] _{k_{i x}=0} \end{aligned}$$

where, $$k_{ix}$$ = *k* sin $$\theta _{i}$$.

And finally, to obtain the PSHE-TD of the field centroid with respect to geometric optic prediction, the following expression is used^20^,

6 $$\begin{aligned} \delta _{\pm }^{H, V}=\frac{\iint \tilde{\textbf{E}}^* i \partial _{k_{r y}} \tilde{\textbf{E}} d k_{r x} d k_{r y}}{\iint \tilde{\textbf{E}}^* \tilde{\textbf{E}} d k_{r x} d k_{r y}}. \end{aligned}$$

In this work, we confine our discussion for *H* polarization state. Considering the first-order approximation of Eq. (5) and using Eqs. (2–6), $\delta _{\pm }^{H}$ is obtained as follows^21^:

7 $$\begin{aligned} \delta _{\pm }^{H}=\mp \frac{k w_0^2 \textrm{R} e\left( 1+r_s / r_p\right) \cot \theta _i}{k^2 w_0^2+\left| \frac{\partial l n r_p}{\partial \theta _i}\right| ^2+\left| \left( 1+r_s / r_p\right) \cot \theta _i\right| ^2}, \end{aligned}$$

Here, $\left| \frac{\partial l n r_p}{\partial \theta _i}\right| ^2$ $\approx$ 0 (discussed in next section), which allows Eq. (7) to be simplified using zeroth-order Taylor series expansion of Eq. (5) and further, solving some mathematical inequalities the following relation can be readily obtained^22,27^:

8 $$\begin{aligned} \delta _{\pm }^{H}=\mp \left( 1+{\text {Re}}\left[ r_s\right] / {\text {Re}}\left[ r_p\right] \right) \cot \theta _{i} / k \end{aligned}$$

If the term $\left| \frac{\partial l n r_p}{\partial \theta _i}\right| ^2$ in Eq. (7) is large, then the first-order approximation of Eq. (5) will be necessary to be considered for increased accuracy^14^. Generally, in such cases, beam waist ‘$w_{0}$’ can be kept sufficiently higher so that the inequality $k^2 w_0^2$ $\gg$ $\left| \frac{\partial l n r_p}{\partial \theta _i}\right| ^2$, which allows obtaining a simplified expression for Eq. (7) with sufficient accuracy for practical applications. From Eq. (8), it is evident that the Fresnel reflection coefficient plays a key role in calculating PSHE-TD. In the following section, device structure will be investigated on maximizing the $\delta _{\pm }^{H}$ with respect to Fresnel reflection coefficients.

## Results and discussion

**Figure 2:**
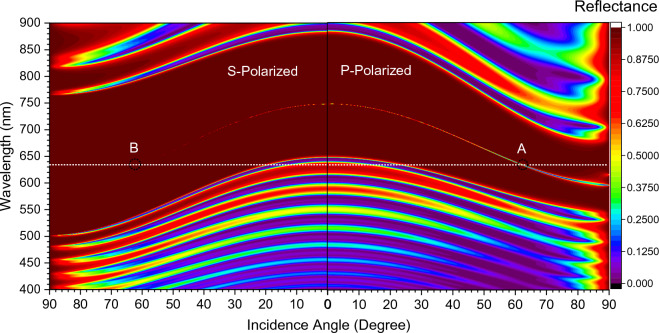
Polarization dependent dispersion analysis of proposed structure: [Substrate|(A,B)$^{N}$ $|$Defect|(A,B)$^{N}$ $|$Air].

**Figure 3:**
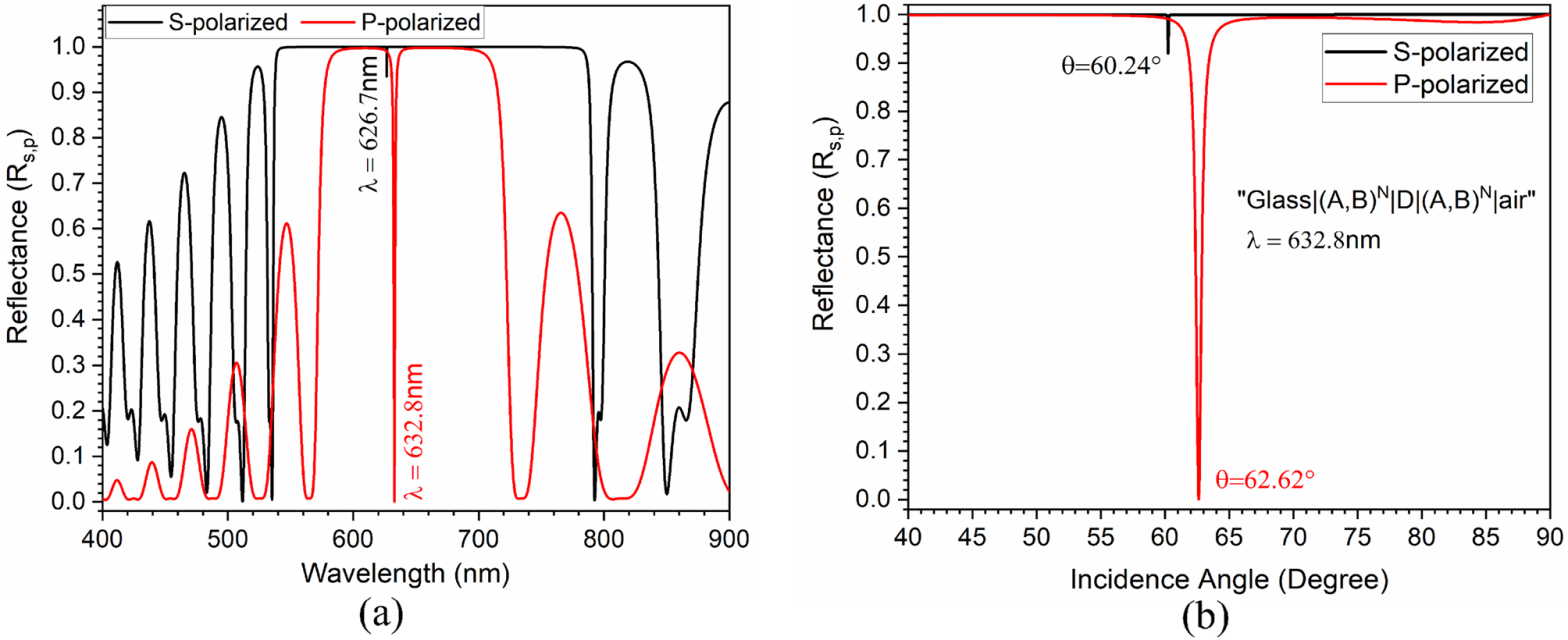
(**a**) Wavelength-dependent reflectance response of the structure at a fixed $\theta _{i}$ of 62.62$^{\circ }$ and (**b**) Angular interrogation at a constant $\lambda$ of 632.8 nm.

The analysis is carried out using the transfer matrix method (TMM), which is used to calculate the structure’s reflection/transmission coefficient. Initially, the angular dispersion analysis of the proposed structure is done to measure its Fresnel reflectance coefficients for both *s*-polarized and *p*-polarized light. Figure 2 shows the angular dispersion diagram of the proposed structure for both *s*-polarization and *p*-polarization states. At a normal incidence, both *s* and *p*-polarization show the generation of a defect state at 750 nm operating wavelength within the photonic bandgap (PBG) of 233 nm (644–877 nm). However, increasing the incidence angle leads to a significant variation in defect mode wavelength for *s*-polarized compared to *p*-polarized incidence light. Therefore, selecting a proper incidence angle and operating wavelength can lead to improvement in $\frac{\mid r_{s}\mid }{\mid r_{p}\mid }$. Initially, the structure is optimized to enhance the PSHE at 632.8 nm operating wavelength, marked by a white line in Fig. 2. However, the same analysis can be carried out at any other user-defined wavelength. For 632.8 nm operating wavelength, two points ‘A’ and ‘B’ are marked in Fig. 2 for further analysis. These points correspond to 62.62° and 60.24° incidence angle, and the corresponding reflectance response is shown in Fig. 3. For an incidence angle of 62.62$^{\circ }$ the structure shows a very low fresnel reflection coefficient for *p*-polarized light, whereas *s*-polarized has a relatively higher reflectance response at 632.8 nm operating wavelength.

**Figure 4:**
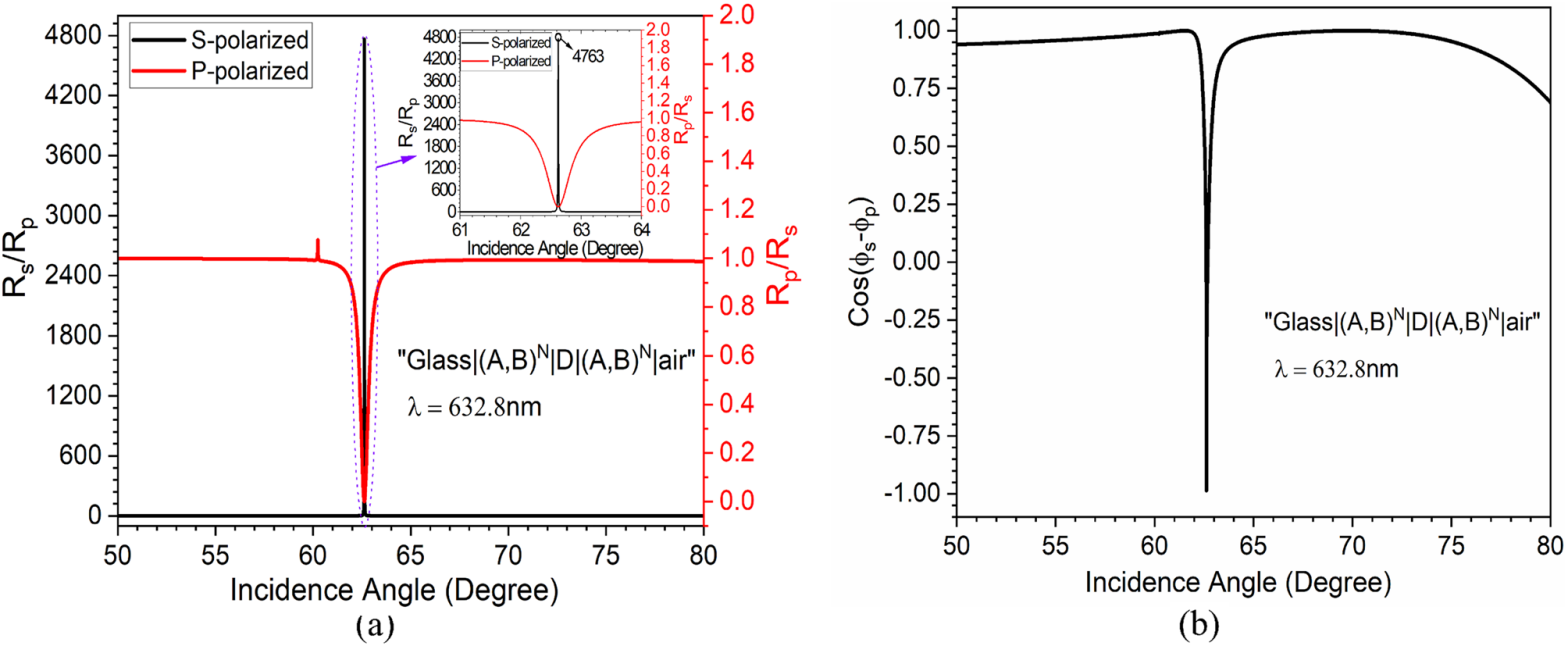
(**a**) Fresnel reflection ratio for the proposed structure at $\lambda$= 632.8 nm and $\theta _{i}$= 62.62$^{\circ }$. (**b**) Phase angle variation with respect to $\theta _{i}$ with a sharp dip observed at $\theta _{i}$= 62.62$^{\circ }$.

Figure 3a shows the wavelength-dependent reflectance response of the proposed structure at a fixed incidence angle of 62.62$^{\circ }$ and in Fig. 3b corresponding angular interrogation at a constant operating wavelength of 632.8 nm is shown. From Fig. 3b, we observe that for a small $\partial \theta _{i}$ at 62.62$^{\circ }$, the term $\left| \frac{\partial l n r_p}{\partial \theta _i}\right| ^2$ $\approx$ 0, which allows for zeroth-order Taylor series expansion of Eq. (5)^14,22,27^ for obtaining $\delta _{\pm }^{H}$. It is clear from Fig. 3 that for 62.62$^{\circ }$ incidence angle, the structure has a much larger reflection for *s*-polarized and a shallow reflection for *p*-polarized light at $\lambda$ = 632.8 nm. The same has been verified by angular interrogation, which is shown in Fig. 3b. Thus, it is expected to have a high ratio of $\frac{\mid r_{s}\mid }{\mid r_{p}\mid }$, which is one of the essential conditions for PSHE-TD enhancement as per Eq. (7). Another essential condition for PSHE-TD enhancement is to have a maximum cosine function (Cos($\phi _{s}$- $\phi _{p}$)). Therefore, the angular dependent reflectance ratio and cosine function is evaluated for both *s* and *p*-polarized light for the proposed structure, which is shown in Fig. 4. The structure exhibits a very high $\frac{\mid r_{s}\mid }{\mid r_{p}\mid }$ of around 4763 for $\theta _{i}$ = 62.62$^{\circ }$ and $\lambda$ = 632.8 nm as shown in Fig. 4a. Whereas, $\frac{\mid r_{p}\mid }{\mid r_{s}\mid }$ is negligible on this wavelength/incidence angle values. The $\frac{\mid r_{s}\mid }{\mid r_{p}\mid }$ shows a large variation at a particularly narrow range of value of $\theta _{i}$ and practically remains insensitive to change in the incidence angle. This behavior is used for sensing-based applications. As $\delta ^H$ is also dependent on $\phi _{s,p}$, the phase difference ($\phi _{s}$- $\phi _{p}$) was analysed. In Fig. 4b, here cos($\phi _{s}$- $\phi _{p})$ value has an abrupt change in magnitude at $\theta _{i}$ = 62.62$^{\circ }$, which is generally observed ($\frac{\mid r_{s}\mid }{\mid r_{p}\mid }$ has large value at this particular $\theta _{i}$).

**Figure 5:**
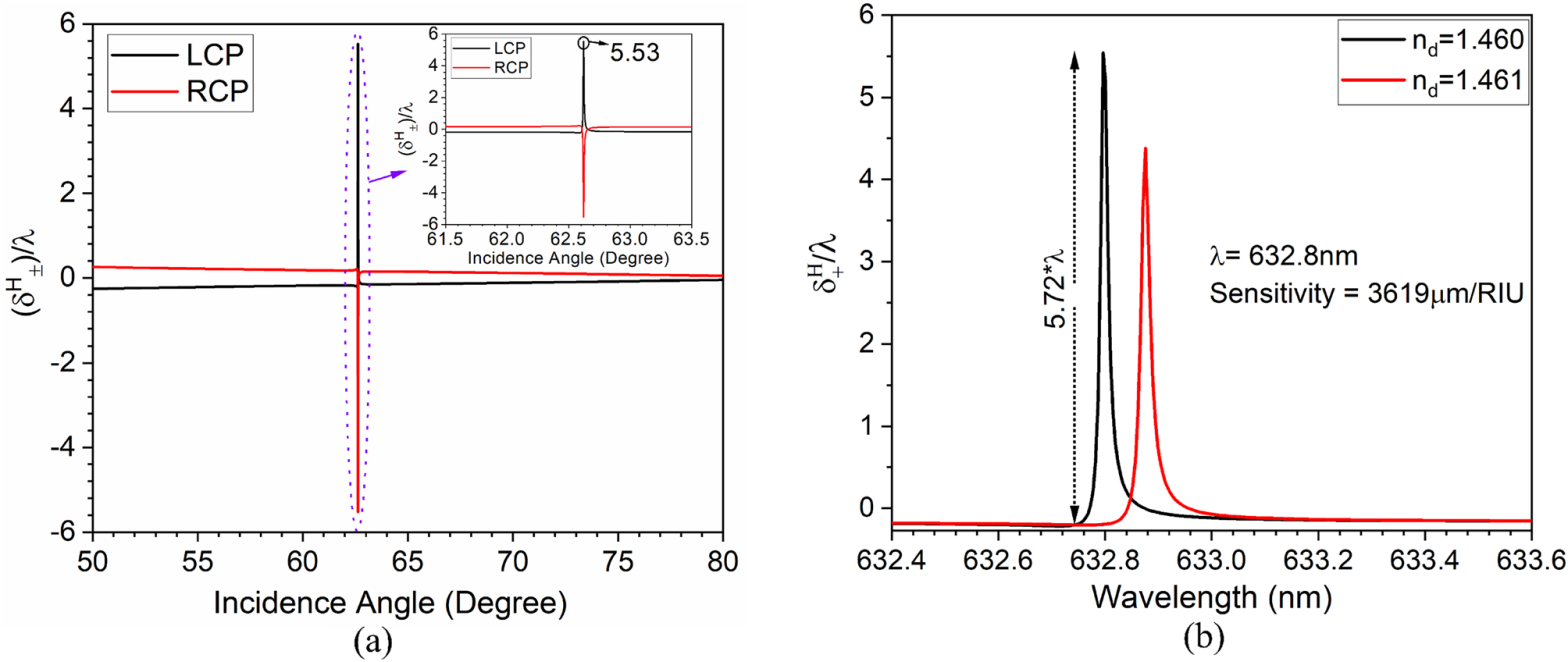
(**a**) Calculated angle dependent wavelength normalized PSHE transverse shift and, (**b**) sensitivity analysis of the proposed structure using PHSE-TD interrogation method.

Finally, angle-dependent PSHE-TD is calculated on the optimized parameters. Figure 5 shows the PSHE-TD for *H* polarized state with respect to $\lambda$. The structure possesses a maximum 5.53$\lambda$ PSHE-TD with the selected parameters as shown in Fig. 5a. The PSHE-TD also exhibits a very narrow full-width-half-maximum (FWHM) of around 0.016 nm. Further, the structure’s sensing capability is demonstrated by considering both wavelength interrogation and PSHE methods. Infiltrating the analyte having varying dielectric constant leads to a change in the effective index of the PhC-cavity. This results in a shift in the operating wavelength, shown in Fig. 5b.

The PSHE-TD ($\delta _{+}^H$) has the same magnitude as $\delta _{-}^H$ based on Eq. (6). Thus, this work considers only $\delta _{+}^H$ for sensitivity analysis. Infiltration of analyte in PhC-cavity leads to a shift in resonance wavelength (or PSHE-TD), which is then measured to calculate the sensitivity. The PSHE-TD interrogation shift sensitivity parameter ($S_{TD}$) at a fixed operating wavelength ($\lambda$) is measured by calculating the shift in PSHE-TD ($\Delta \delta _{+}^H$) for a corresponding difference in the refractive index of the infiltrated analyte in the PhC-cavity structure. Thus,

9 $$\begin{aligned} \left. S_{T D}^H\right| _{\lambda = fixed}=\frac{\Delta \delta _{+}^H}{\Delta n_d} \end{aligned}$$

Since the structure is very sensitive to a minute refractive index change, therefore, the PSHE-TD structure-based shift sensitivity is calculated by considering a 0.001 variation in the refractive index of PhC-cavity (1.460 to 1.461). This gives a shift in PSHE-TD ($\Delta \delta _{+}^H$) of around 5.72$\lambda$ (5.53$\lambda$ at 1.460 and − 0.1928$\lambda$ at 1.461) for a corresponding index variation ($\Delta$ $n_d$) of 0.001. This gives the average PSHE-TD shift sensitivity ($S_{T D}^H$) of around 3619 μm/RIU as shown in Fig. 5b. Moreover, the structure shows a figure of merit (FOM = $\frac{S_{T D}^H}{\lambda _{1/2}}$) of around 2.26$\times 10^{8}$ $\hbox {RIU}^{-1}$.

**Figure 6:**
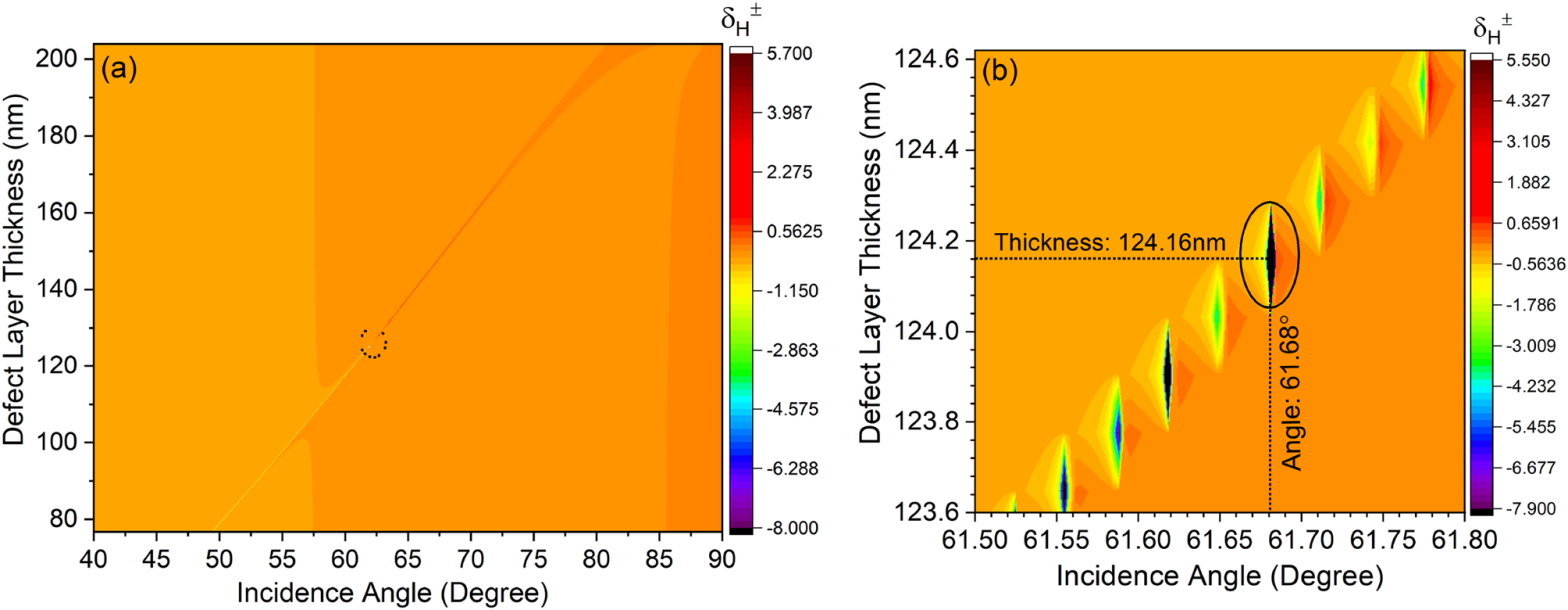
Defect layer thickness variation with respect to incidence angle.

Further, the wavelength interrogation sensitivity parameter ($S_{\lambda }$) at a fixed $\mid \delta _{max}^H \mid$ is measured by calculating the shift in resonance wavelength ($\Delta \lambda$) for a corresponding difference in the refractive index of the infiltrated analyte ($\Delta n_d$) in the PhC-cavity structure. Thus,

10 $$\begin{aligned} \left. S_{\lambda }\right| _{ \delta _{max}^H =fixed}=\frac{\Delta \lambda }{\Delta n_d} \end{aligned}$$

**Figure 7:**
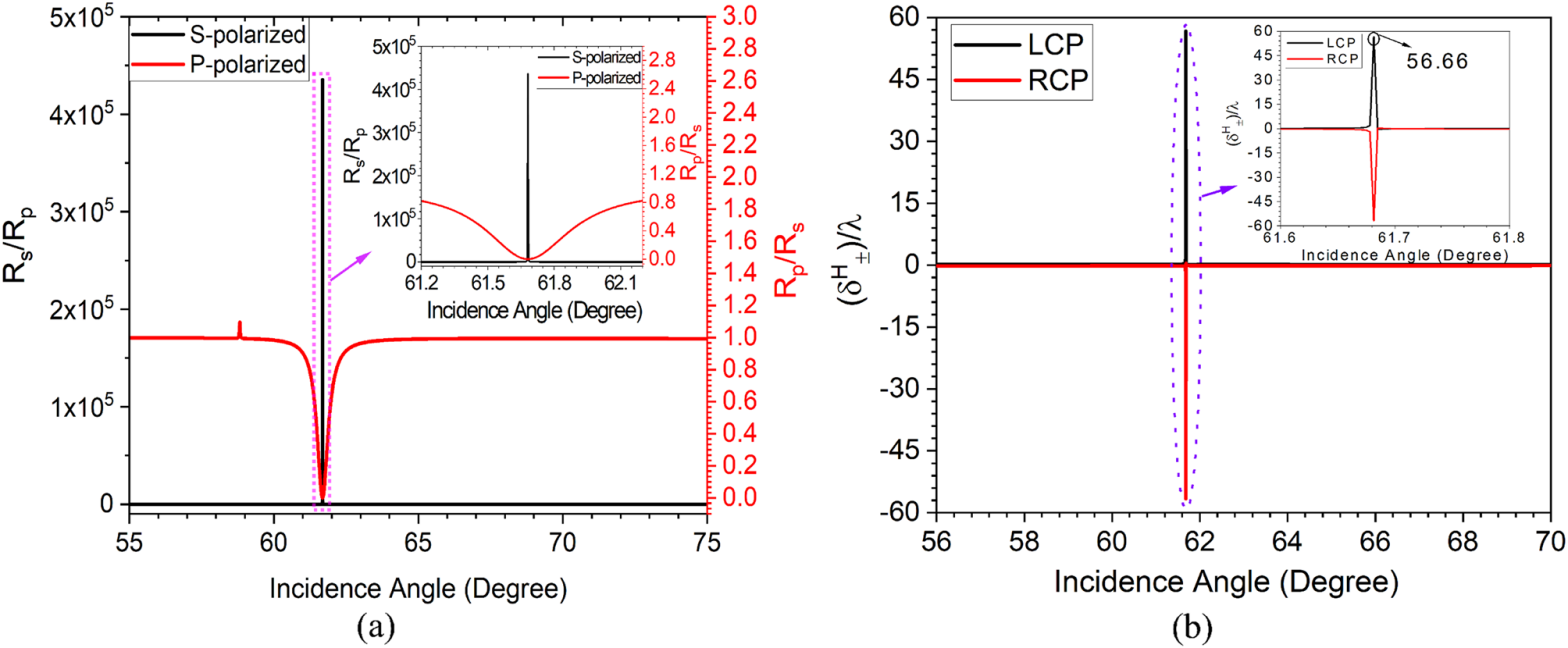
(**a**) Fresnel Reflection ratio for the proposed structure at $\lambda$= 632.8 nm and $\theta _{i}$= 61.68$^{\circ }$, and (**b**) enhanced PSHE after optimization in defect layer thickness.

This gives an average sensitivity ($S_{\lambda }$) $\approx$ 79 nm/RIU for the wavelength interrogation method. Although the proposed structure shows a high PSHE-TD of around 5.53$\lambda$ with sensitivity in $S_{\lambda }$ and $S_{T D}$ of about 79 nm/RIU and 3619 μm/RIU, respectively. However, these can further be enhanced by optimizing the $D_{d}$ and obtaining the corresponding optimized resonance angle ($\theta _{r_{o}}$) for PSHE-TD maximization. This can be accomplished by optimizing the exceptional points for the proposed structure. Therefore, the impact of defect layer thickness is further investigated. Figure 6a shows the defect layer thickness-dependent PSHE characteristics of the proposed structure. The structure shows a good inversion property of PSHE near the resonance angle, shown by a black circle in Fig. 6a. At the inversion point, the structure shows exceptional points with high PSHE-TD. The analysis demonstrates a maximum PSHE for the proposed structure at an optimized defect layer thickness $D_{d_{o}}$ of 124.16 nm and corresponding $\theta _{r_{o}}$ of 61.68$^{\circ }$ as illustrated in Fig. 6b. Further, considering Eqs. (5–7), all the parameters (e.g., $\frac{\mid r_{s}\mid }{\mid r_{p}\mid }$, Cos($\phi _{s}$- $\phi _{p}$), and $\delta _{\pm }^{H}$) including the PSHE-based sensitivity are re-evaluated for the newly optimized exceptional parameter and are shown in Fig. 7. Here, the zeroth-order Taylor series expansion is again used to calculate the PSHE-TD (because of $\left| \frac{\partial l n r_p}{\partial \theta _i}\right| ^2$ $\approx$ 0). The structure shows a very high $\frac{\mid r_{s}\mid }{\mid r_{p}\mid }$ of around 4.35$\times 10^{5}$, which is around 91 times higher than the previous value of Fig. 4a, as shown in Fig. 7a . This gives a PSHE shift ($\delta _{\pm }^{H}$) of about 56.66$\lambda$ at $\theta _{r_{o}}$ = 61.68$^{\circ }$, which is around 924% higher than Fig. 5a value and is represented in Fig. 7b. Moreover, the structure exhibits a very narrower full-width-half-maximum (FWHM) of around 0.005 nm.

**Figure 8:**
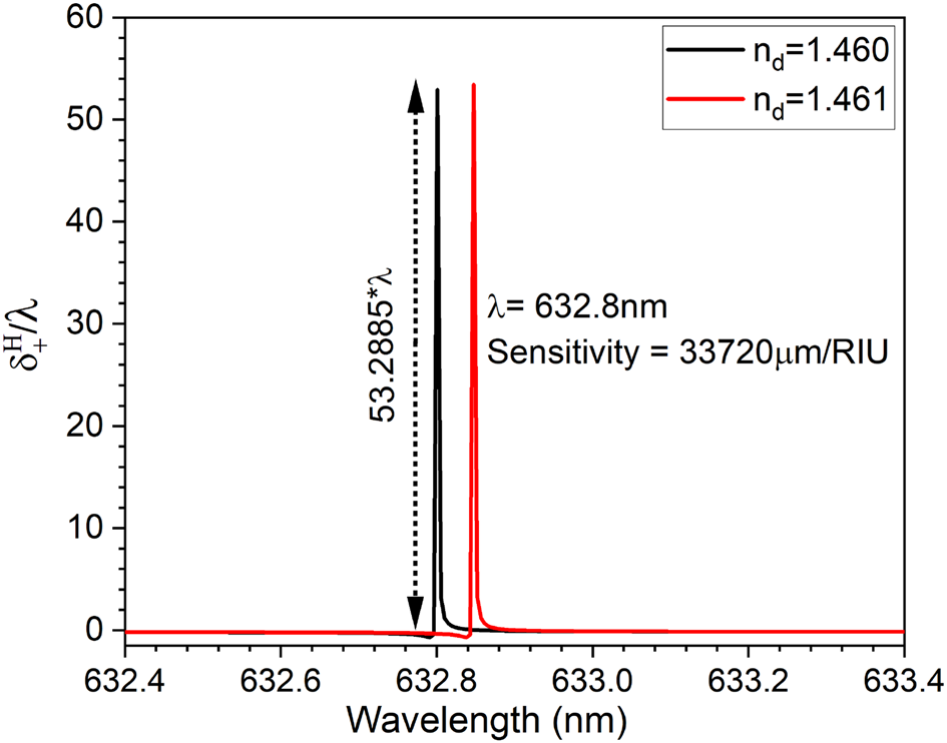
Optimized PSHE-TD sensitivity ($S_{T D}^H$) of about 33,720 μm/RIU.

Moreover, the obtained PSHE-TD shift is much higher than recently reported values^14,24,34^. Considering these optimized exceptional parameters, the structure sensing capability is re-evaluated. The structure sensitivity to a small refractive index change is utilized to obtain the PSHE-TD shift sensitivity by considering a 0.001 variation in the refractive index of PhC-cavity (1.460 to 1.461). This gives a PSHE-TD ($\Delta \delta _{+}^H$) of around 53.2885$\lambda$ (52.925$\lambda$ at 1.460 and − 0.3635$\lambda$ at 1.461) for a corresponding index variation of 0.001. This gives the average PSHE-TD shift sensitivity ($S_{T D}^H$) of around 33,720 μm/RIU ($\approx$ 8.31 times the value before optimization) as shown in Fig. 8. Moreover, the structure exhibits a FOM of around 6.7$\times 10^{9}$ $\hbox {RIU}^{-1}$. Similarly, the analytical results exhibit an average wavelength sensitivity of around 46 nm/RIU for wavelength interrogation. Finally, the structure’s PSHE-based refractive index sensitivity is compared with recently reported structures and is shown in Table 1. Compared to recently reported PSHE sensors, the proposed structure shows substantially better PSHE-TD performance, leading to a much higher sensitivity. The structure also shows its capability to detect an analyte having a very small index variation of 0.001 in a much wider refractive index range (1.0 - 1.5). Moreover, the proposed structure can easily be fabricated using simpler spin/dip coating and deposition techniques^35,36^ and PSHE-TD characterization can be done using weak measurement method^37,38^.

**Table 1:** Recently proposed PSHE based refractive index sensor values.

Structure	Materials	Wavelength	Sensitivity (μm/RIU)	PSHE-TD	RI sensing range	Year
SPR	Sodium/PMMA/graphene	1200 nm	1844.9	0.325 μm	1.3195–1.346	2021^25^
SPR	Au/antimonene	633 nm	5468	61.67 μm	1.330–1.335	2021^39^
SPR	Au/graphene	633 nm	$10^5$ (amplified)	$\approx$ 0.09 μm	1.330–1.335	2018^27^
SPR	Fused Ag/Au	632.8 nm	6602	5.34 μm	1.458–1.459	2018^14^
PTS cavity	Epsilon-near-zero material	632.8 nm	8762	4.74 μm	1.000–1.001	2021^34^
LMR	Indium tin oxide/water	1151.9 nm	13,500	13.28 μm	1.333–1.334	2022^24^
1D-PhCR	$$\begin{array}{c}\mid ({ A},\,{ B})^{10} \mid { D}\mid ({ A},\,{ B})^{10} \mid \\ \text {A,D}=\text {SiO}_{\text {2}},\text {B}=\text {Si}_{\text {3}}\text {N}_{\text {4}} \end{array}$$	632.8 nm	33,720	35.85 μm	1.460–1.461	$$\begin{array}{c}\text {This} \\ \text {work} \end{array}$$

## Conclusion

This work presents a theoretical and analytical analysis for utilizing a PhC nano-photonic resonator (1D-PhCR) structure for enhanced photonic spin Hall effect generation. The structural parameters are optimized to enhance the PSHE-TD significantly. A transverse PSHE-based shift of 56.66 times the operating wavelength and PSHE-based refractive index sensitivity of 33,720 μm/RIU is reported in this work. This is attributed to the optimized exceptional points having a particular angle of incidence (61.68$^{\circ }$) and defect layer thickness (124.16 nm). Compared to recently reported PSHE sensors, the proposed structure shows substantially better PSHE-TD performance, leading to a much higher sensitivity. The structure also shows its capability to detect an analyte having a very small index variation of 0.001 in a much wider refractive index range (1.0–1.5). Due to the purely dielectric material-assisted PhC resonator configurations used in this work, more low-cost and simple structure-assisted devices can be designed. Additionally, because of significantly improved PSHE-TD, the development of low-cost PSHE-based devices for commercial applications is envisaged.

## Data Availability

The data may be obtained from the corresponding author upon reasonable request.
